# Identification and Characterization of σ^S^, a Novel Component of the *Staphylococcus aureus* Stress and Virulence Responses

**DOI:** 10.1371/journal.pone.0003844

**Published:** 2008-12-03

**Authors:** Lindsey N. Shaw, Catharina Lindholm, Tomasz K. Prajsnar, Halie K. Miller, Melanie C. Brown, Ewa Golonka, George C. Stewart, Andrej Tarkowski, Jan Potempa

**Affiliations:** 1 Department of Biology, University of South Florida, Tampa, Florida, United States of America; 2 Department of Rheumatology & Inflammation Research, University of Goteborg, Goteborg, Sweden; 3 Department of Microbiology, Faculty of Biotechnology, Jagiellonian University, Kraków, Poland; 4 Department of Biochemistry & Molecular Biology, University of Georgia, Athens, Georgia, United States of America; 5 Department of Veterinary Pathobiology and Bond Life Sciences Center, University of Missouri, Columbia, Missouri, United States of America; Centre for DNA Fingerprinting and Diagnostics, India

## Abstract

*S. aureus* is a highly successful pathogen that is speculated to be the most common cause of human disease. The progression of disease in *S. aureus* is subject to multi-factorial regulation, in response to the environments encountered during growth. This adaptive nature is thought to be central to pathogenesis, and is the result of multiple regulatory mechanisms employed in gene regulation. In this work we describe the existence of a novel *S. aureus* regulator, an as yet uncharacterized ECF-sigma factor (σ^S^), that appears to be an important component of the stress and pathogenic responses of this organism. Using biochemical approaches we have shown that σ^S^ is able to associates with core-RNAP, and initiate transcription from its own coding region. Using a mutant strain we determined that σ^S^ is important for *S. aureus* survival during starvation, extended exposure to elevated growth temperatures, and Triton X-100 induced lysis. Coculture studies reveal that a σ^S^ mutant is significantly outcompeted by its parental strain, which is only exacerbated during prolonged growth (7 days), or in the presence of stressor compounds. Interestingly, transcriptional analysis determined that under standard conditions, *S. aureus* SH1000 does not initiate expression of *sigS*. Assays performed hourly for 72h revealed expression in typically background ranges. Analysis of a potential anti-sigma factor, encoded downstream of *sigS*, revealed it to have no obvious role in the upregulation of *sigS* expression. Using a murine model of septic arthritis, *sigS*-mutant infected animals lost significantly less weight, developed septic arthritis at significantly lower levels, and had increased survival rates. Studies of mounted immune responses reveal that *sigS*-mutant infected animals had significantly lower levels of IL-6, indicating only a weak immunological response. Finally, strains of *S. aureus* lacking *sigS* were far less able to undergo systemic dissemination, as determined by bacterial loads in the kidneys of infected animals. These results establish that σ^S^ is an important component in *S. aureus* fitness, and in its adaptation to stress. Additionally it appears to have a significant role in its pathogenic nature, and likely represents a key component in the *S. aureus* regulatory network.

## Introduction

*Staphylococcus aureus* is a major human pathogen that is a leading agent of both nosocomial and community acquired infections. It is both a highly successful and dangerous pathogen that poses a significant threat to public health due to the increased prevalence of antibiotic resistant strains, such as methicillin-resistant *S. aureus* (MRSA) [1]–[4]. The appearance in recent years of true vancomycin-resistant MRSA [5]–[9] presents us with a frightening prospect of a return to the days of pre-antibiotic medicine, where the vast majority of staphylococcal bloodstream infections proved fatal. One of the overwhelming reasons that *S. aureus* is such a successful and diverse pathogen is the arsenal of virulence determinants encoded within its genome, which include hemolysins, toxins, adhesins and other exoproteins, such as proteases, staphylokinase and protein A [10], [11]. These damaging virulence factors are subject to multi-level and multi-factorial regulation, both temporally and spatially, in response to the environments encountered during growth [11]. This responsive and adaptive nature is thought to be central to the disease-causing ability of the organism, and is largely the result of the multiple regulatory mechanisms it employs in gene regulation.

The large and wide reaching regulatory network employed by *S. aureus* encompasses a variety of common bacterial regulatory mechanisms, including two-component regulators, DNA binding proteins, regulatory RNAs, sigma factors and a quorum sensing system. There are thought to be sixteen two-component systems in *S. aureus*, including those that are responsible for the modulation of autolysis (ArlRS, LytRS), virulence (AgrAC, SaeRS) cell wall synthesis/drug resistance (GraRS, VraSR), and the sensing of external iron (HssRS) and oxygen (SrrRS) [12]–[18]. In addition there is a central, master regulator of virulence, the Agr system, which is encoded by a four-gene locus that regulates pathogenesis, and the shift from localized to invasive phenotypes [19]–[21]. Further regulators exist, including the 12 members of the SarA family of DNA binding proteins [22], several of which have been shown to be important in virulence factor synthesis (SarA, Rot, SarT) [23]–[25]. There are also three metal-dependent DNA binding proteins encoded within the *S. aureus* genome, two of which (Fur and PerR) are required for the survival of *S. aureus* in animal models of infection [26].

*S. aureus* also has 3 known sigma factors: a housekeeping sigma factor, σ^A^, originally described by Deora and Misra [27], and two alternative sigma factors, σ^B^ and σ^H^ [28], [29]. Of these three, σ^B^ is by far the most widely studied, the effects of which are apparent in a variety of cellular processes, including oxidative stress resistance, pigmentation, protein secretion, biofilm formation, drug resistance, adaptation to stress and the progression of disease [30]–[32]. Indeed, strains of *S. aureus* lacking a functional σ^B^ are pleiotropically altered at the phenotypic level, and demonstrate reduced virulence in *in vivo* models of animal infection [30], [33]. σ^A^, encoded by the *plaC* gene, was first identified over a decade ago based on its homology with σ^A^ from *B. subtilis* [27]. It is analogous to other primary sigma factors in that it is essential for growth, and controls much of the day-to-day house-keeping transcription. Documentation of a third sigma factor, σ^H^, in *S. aureus* recently appeared in a study by Morikawa *et al*. [28]. Here it was shown that *S. aureus* possesses a homologue of the genetic competence sigma factor, σ^H^, from *B. subtilis*.

While the primary sigma factor directs much of the transcription during growth, most organisms possess alternative sigma factors that direct the transcription of specific regulons during unusual physiological conditions. ECF, or extra-cytoplasmic function, sigma factors form a distinct and diverse subfamily within this class of regulators that often share distant or divergent identity with other known σ factors. As a group, they are by far the most numerate of the sigma factor families [34], [35], with *Streptomyces coelicolor* possessing more than 50 such elements within its genome. Other organisms, including *Mycobacterium tuberculosis*, *Pseudomonas aeruginosa* and *Bacillus anthracis* encode 10 or more such factors [34]. They have been identified in a variety of Gram-negative and Gram-positive organisms, and have been shown to have wide-ranging and varied roles in cellular physiology. These include the adaptation to: antimicrobial compounds, salt stress, elevated or reduced growth temperatures, acidic pH, detergents, oxidative stress, disulphide stress, iron starvation, osmotic stress, carbon and nitrogen stress, high pressure and light [36]–[45]. More importantly however, as the number of ECF-sigma factors identified grows, attention is turning to their often considerable roles in the virulence of pathogenic organisms [46].

Unusually, *S. aureus* seemingly achieves its versatile and adaptive nature with only a limited selection of sigma factors. So far only three have been documented, and only one of these (σ^B^) has been shown to have a role in cellular adaptation and virulence. In this work we describe the characterization of a fourth *S. aureus* sigma factor, an apparent ECF-sigma factor, which is seemingly involved in cellular fitness and the adaptation to stress. Additionally it appears to have a significant role in the pathogenic nature of *S. aureus*, and likely represents an additional, key component in the regulatory network of this organism.

## Results

### Identification of SACOL1827 as a putative ECF-sigma factor

During work in our laboratory on the membrane proteases of *S. aureus,* we generated a mutation in RseP. Multiple publications on RseP proteases in *E. coli*, *B. subtilis* and *Pseudomonas aeruginosa* demonstrate that they commonly serve to cleave the anti-sigma factors of extra-cytoplasmic function (ECF)-sigma factors [47]–[53]. As it has previously been proposed by Helmann that the genome of *S. aureus* likely contains an ECF-sigma factor [34], we undertook an exploration of the *S. aureus* genome so as to determine whether an as yet unidentified ECF sigma factor was present. Using the protein sequence of the 7 known *B. subtilis* ECF-sigma factors, a novel protein (SACOL1827) bearing homology to the ECF-sigma factors σ^M^ and σ^YlaC^ was discovered in the *S. aureus* genome (Table 1). BLAST analysis with this protein sequence revealed homology with other ECF-sigma factors from a variety of organisms (Table 1). The gene coding this protein is present in the genome of all of the sequenced strains of *S. aureus*. Equally, it is present in the four other sequenced Staphylococcal genomes: *S. epidermidis* ATCC 12228 and RP62A, as well as *S. haemolyticus* and *S. saprophyticus*. Our initial investigations of SACOL1827, using *in silico* protein analysis, demonstrated the presence of both regions 2 and region 4 of σ^70^. Further, *in silico* protein folding analysis (using the 3D-JIGSAW, FUGUE and PHYRE databases) generated strong homology scores for both of these regions (between 95–100% certainty for region 2, and 90–95% certainty for region 4). Overall our predictive protein folding and modeling analyses returned a probability value of p = <0.001 for σ^S^ against the founding-member of the ECF-sigma factors, σ^E^ of *E. coli*.

**Table 1:** Blast analysis for proteins homologous to SACOL1827 from *Staphylococcus aureus*.

Organism	Assignment	Identities	Positives
		No. of identical residues/total no. of aligned residues	No. of similar residues/total no. of aligned residues
*B. subtilis*	σ^M^ ECF σ factor	33/164 (20%)	73/164 (44%)
*B. subtilis*	σ^YlaC^ ECF σ factor	29/143 (20%)	69/143 (48%)
*Idiomarina loihiensis*	ECF σ factor	36/128 (28%)	69/128 (53%)
*B. thetaiotaomicron*	ECF σ factor	36/134 (26%)	72/134 (53%)
*Pseudoalteromonas atlantica*	ECF σ factor	35/121 (28%)	62/121 (51%)
*B. cereus*	σ^M^ ECF σ factor	31/108 (28%)	55/108 (50%)
*V. parahaemolyticus*	ECF σ factor	37/140 (26%)	70/140 (50%)
*Oceanobacillus iheyensis*	ECF σ factor	35/121 (28%)	59/121 (48%)
*C. botulinum*	BotR/A σ^70^ family	33/147 (22%)	78/147 (53%)

A common observance of ECF-family proteins is that the genes encoding the sigma factors are contiguous to a coding region specifying an anti-sigma factor. Analysis of the SACOL1827 locus revealed a putatively transcriptionally-linked downstream gene (SACOL1828) that is separated from SACOL1827 by 112 bp. SACOL1828 is a conserved hypothetical protein with no discernable homology to other proteins within the databases, other than its direct homologues in staphylococci. *In silico* analysis determined that these two genes are found clustered in this arrangement in all of the sequenced *S. aureus* genomes (including the RF122 bovine mastitis strain); in *S. epidermidis* ATCC 12228 and RP62A; and in *S. haemolyticus* and *S. saprophyticus*. Commonly the anti-sigma factors of ECF-sigma factors possess membrane associated domains, however analysis of SACOL1828 using a Kyte-Doolittle hydrophobicity plot revealed no such region. Interestingly, some ECF anti-sigma factors possess an H(XXX)C(XX)C motif, as is the case in *Streptomyces coelicolor* and *Mycobacterium tuberculosis* [54]–[56]. SACOL1828 bears a similar sequence of H(LETN)C(VFH)C, which correlates well with that found in other organisms.

### Biochemical characterization of SACOL1827 reveals it to be a sigma factor

**Figure 1:**
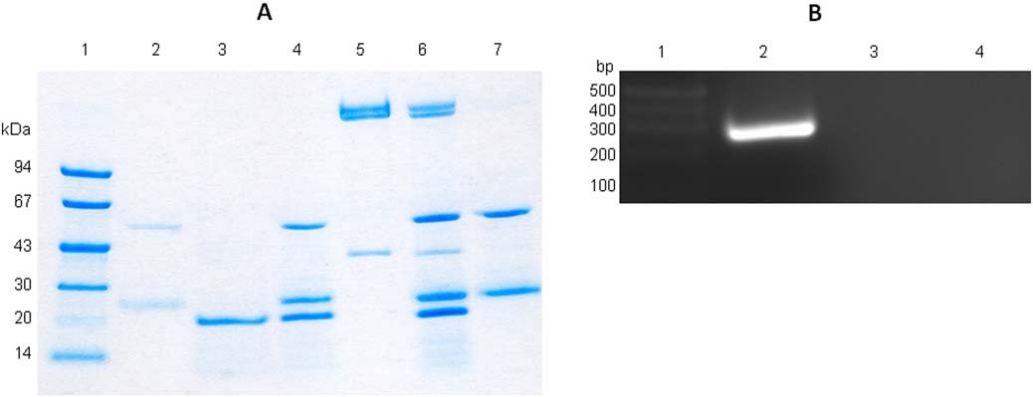
Biochemical characterization of the SACOL1827 protein. (A) Pulldown Assay showing association of SACOL1827 with core-RNAP. Lane order: L1, LMW Markers; L2, Monoclonal Anti-poly Histidine–Agarose antibody (with beads); L3, SACOL1827; L4, SACOL1827 (with beads); L5, core-RNAP; L6, SACOL1827 + core-RNAP (with beads); L7, core-RNAP (with beads); L8, HMW Markers. (B) Transcription run-off assay. Lane order: L1, DNA size markers; L2, transcription run-off conducted with core-RNAP + purified SACOL1827; L3, transcription run-off conducted with core-RNAP only; L4, transcription run-off conducted with purified SACOL1827 only.

Sigma factors bind to core RNAP in a reversible way in order to induce transcription. To test the ability of *S. aureus* SACOL1827 to bind to core-RNAP we generated recombinant protein using standard *E. coli* overexpression techniques, and the 6HIS-tagging vector pET24d (Novagen), as described previously [57]. Pulldown assays were then performed using the purified protein and *E. coli* core-RNAP (Epicentre). Recombinant SACOL1827 was coupled to Ni-NTA agarose beads (via the HIS tag), followed by the addition of core RNAP. Beads were then washed, resuspended in sample buffer and loaded onto a SDS-PAGE gel. As a control, this analysis was repeated in parallel omitting purified SACOL1827. We determined that in the absence of SACOL1827, core-RNAP was unable to bind the Ni-NTA beads, whilst in the presence of SACOL1827 core-RNAP copurified upon elution (Fig. 1A).

Another common feature of ECF-sigma factors is that they have a role in the autoregulation of their own expression. With this in mind we decided to test the ability of SACOL1827 to initiate transcription from its own locus by transcriptional run off analysis. Core-RNAP was preincubated with purified SACOL1827 protein for 15 mins at 4°C, before the addition of an 1168 bp DNA-fragment containing the *sigS* coding region and 945 bp of upstream sequence. After further incubation (at 37°C for 15mins) transcription was initiated by the addition of rNTPs, and allowed to proceed for 30 mins. The mixture was then cleaned via two acid-phenolchloroform extractions (to remove DNA contamination), and an isopropanol precipitation. The purified mRNA transcript was then subject to a 1-step RT-PCR reaction with primers internal to the SACOL1827 coding region (104 bp from the initiation codon to 137 bp from the termination codon). This experiment was repeated with controls, where either purified SACOL1827 protein or core-RNAP was omitted from the reaction mixture. The RT-PCR reactions were then resolved on a 2% agarose gel (Fig. 1B), and revealed that only the SACOL1827-core-RNAP complex lane yielded the expected DNA fragment of 274 bp. The 2 control lanes demonstrated an absence of bands, indicating that the acid-phenolchloroform extractions effectively removed the template DNA. As the SACOL1827-core-RNAP complex is capable of specifically initiating transcription, we termed the SACOL1827 gene *sigS*, and its resultant protein σ^S^.

### Analysis of a *sigS* mutant reveals a role for σ^S^ in the *S. aureus* stress response

**Figure 2:**
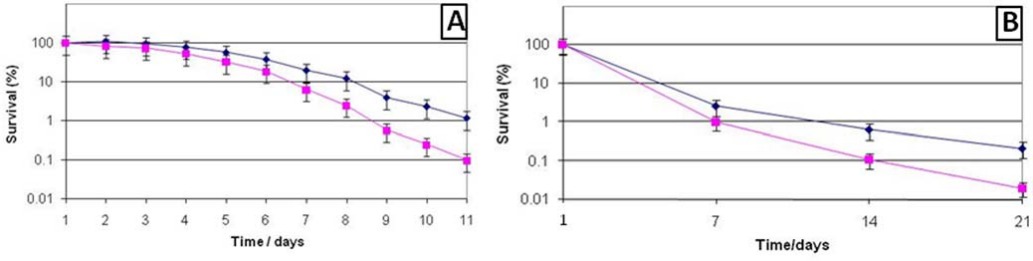
Long term survival of the *sigS* mutant. The SH1000 *sigS* (▪) mutant, along with its parental strain (⧫), were grown in TSB for 11 (A) or 21 (B) days. CFU/ml were determined at the specified intervals and are expressed as percentage survival.

A common role of ECF-sigma factors is to protect bacterial cells against external stress. In order to investigate if *sigS* has such a purpose in *S. aureus* we created a SH1000 *sigS*::*tet* insertionally inactivated mutant strain. Growth of the mutant was compared to the wild-type and found to be indistinguishable in TSB media under standard conditions (data not shown). However when long term survival experiments were conducted (11 days, aerobic growth, standard conditions) the *sigS* mutant showed a more pronounced decrease in viability than the parental strain (Fig. 2A). The *sigS* mutant strain lost viability at a consistently greater rate than that of the parental strain, an effect that became more pronounced as the experiment was prolonged. In order to assess the long term implications of this, the mutant and parental strain were subjected to starvation survival experiments over a period of 3 weeks (Fig. 2B). As with the 11 day experiment, the mutant strain had a decreased viability during long term starvation when compared to the parental strain.

**Figure 3:**
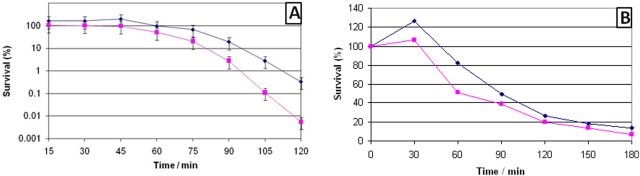
(A) Death curves of the *sigS* mutant and parental strain. (A), The effect of elevated temperature (55°C) on cellular viability. Exponentially growing SH1000 (⧫) and the *sigS* mutant (▪) were shifted from growth at 37°C to growth at 55°C, and viabilities were determine by CFU/ml at the time intervals specified. The standard deviation of five replicate cultures is shown in the form of error bars. (B) Triton X-100 induced lysis of the *sigS* mutant and its parental strain. SH1000 (⧫) and the *sigS* mutant (▪) were lysed using 0.05% Triton X-100 and the CFU/ml determined at the time intervals specified.

ECF-sigma factors in a number of organisms have been shown to be important in the response to elevated temperature stress [38], [44], [58]. Therefore we tested the ability of the *sigS* mutant to grow at elevated temperatures (40°C and 45°C), and to survive heat shock (exponential cultures placed at 55°C for 15 mins before being returned to growth at standard conditions). In each case the *sigS* mutant strain responded to alterations in heat in a manner akin to that of the parental strain (data not shown). However, when we tested the viability of exponentially growing cultures subjected to growth at 55°C, it was found that over a 2 hour period the *sigS* mutant was more sensitive to killing by the elevated temperature (Fig 3A). Following this, further death curves were performed using the *sigS* mutant and its parental strain, in the presence of 0.4 mg ml^−1^ penicillin G, 50 µg ml^−1^ lysostaphin and 0.05% Triton X-100. In the case of lysostaphin and penicillin G no obvious difference was determined between the *sigS* mutant and its parental strain. However, when Triton X-100 was used as a lytic agent, the *sigS* mutant lysed at a quicker initial rate than that of the parental strain (Fig. 3B). This early variations in lysis was not borne out through the entire experimental time course, as the parent and mutant strain reached equivalent levels of survival after approximately 2 h.

Further to these experiments we set out to explore the role of *sigS* in *S. aureus* physiology by subjecting the mutant strain to growth analysis under a variety of different stress conditions. Disk diffusions assays were conducted with SH1000 and the *sigS* mutant in the presence of oxidative stress inducing compounds (30% H_2_0_2_, 80% cumene hydroperoxide, 500mM diamide, 2M methyl viologen, 1% menadione, 100mM plumbagin, 400 mg ml^−1^ pyrogallol), nitric oxide stress inducing compounds (100mM sodium nitroprusside), detergent stress (10% SDS, 10% Triton X-100), acid (12M HCl) and alkali stress (6M NaOH), alcohol stress (95% ethanol) and the antibiotics bacitracin (2 mg ml^−1^), vancomycin (2 mg ml^−1^), penicillin G (5 mg ml^−1^) and puromycin (20 mg ml^−1^). In each case no alteration in the zones of growth inhibition were observed (data not shown). The mutant and parental strain were tested further by growing them separately in liquid media containing 1 M and 2.5 M NaCl, 20 mM Glucose, and acidic and alkaline adjusted media (pH 5, with HCl; and pH 9, with NaOH). Again no alterations in growth were detected between the wild-type and mutant strain (data not shown).

### Competitive growth analysis reveals the σ^S^ mutant has a decreased fitness for survival

**Figure 4:**
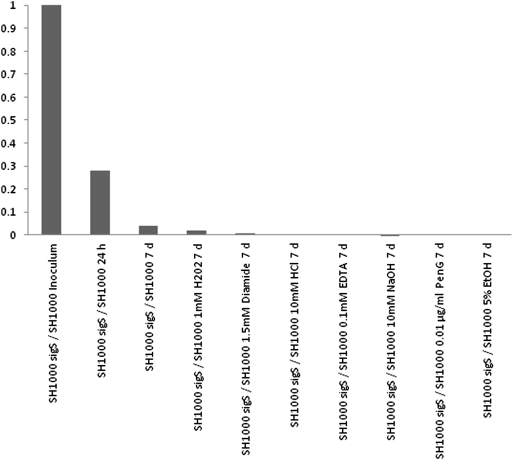
Competitive growth analysis of the *sigS* mutant and its parental strain. SH1000 and its *sigS* mutant derivative were cocultured in TSB or TSB containing subinhibitory concentrations of: hydrogen peroxide (1 mM), diamide (1.5 mM), HCl (10 mM), NaOH (10 mM), EDTA (0.1 mM), penicillin G (0.01 µg ml^−1^) or ethanol (5%). The competitive index (CI) was determined for each strain after the respective growth periods and represents the relative proportion of the two strains after inoculation at a 1∶1 ratio. Data is representative of at least 3 independent cultures.

Competitive growth experiments were undertaken to assess the viability of the SH1000 σ^S^ mutant when grown in coculture experiments with its parental strain SH1000. These experiments are facilitated by the fact that the σ^S^ mutant is marked with a tetracycline resistance cassette; thus plating dilutions of the coculture on both TSA (Tryptic Soy Agar) and TSA containing tetracycline, allows derivation of exact colony counts for each strain, and thus calculation of the competitive index (CI). What was found was that SH1000 inoculated with the σ^S^ mutant in a 1∶1 ratio resulted in a 1∶0.28 ratio after 24 hours growth (Fig. 4). The mutant was even further impaired in its competitive abilities against the parental strain after 7 days of growth, resulting in a growth ratio of 1∶0.04. As ECF-sigma factors commonly serve to protect the cell during times of stress we hypothesized that *sigS* mutant would show additional decline in coculture experiments with the parent when grown in the presence of sub-inhibitory concentrations of stress-inducing compounds. Indeed, whilst little variation from non-stressed conditions was observed after 24 hours growth, significant differences were observed after 7 days growth. When the experiments were repeated using the oxidative stress inducing chemicals hydrogen peroxide (1 mM) and diamide (1.5 mM) 7 day ratios were found to be 1∶0.02 and 1∶0.01, respectively. Additionally when the pH was altered in coculture flasks using HCl (10 mM) or NaOH (10 mM) further declines were seen, yielding 7 day ratios of 1∶0.005 and 1∶0.0006, respectively. Similarly coculture experiments using the metal ion chelator EDTA (0.1 mM) produced 7 day ratios of 1∶0.003. Finally, and most dramatically, experiments using penicillin G (0.01 µg ml^−1^) and ethanol (5%) yielded no detectable *sigS* mutant colonies after 7 days of growth with the parental strain.

### Transcription profiling analysis of *sigS* expression

**Figure 5:**
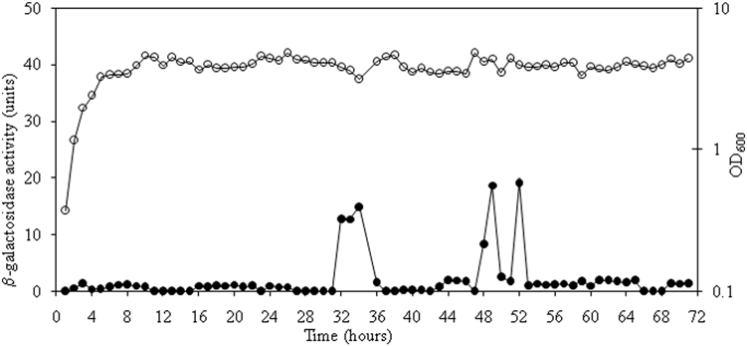
Expression analysis of *sigS* using a *lacZ* reporter-fusion strain. An SH1000 *sigS*-*lacZ* strain was grown for 72 hours, with samples withdrawn every hour to quantify the relative amount of *sigS* expression (•). The OD600 of the strain was also measured at each time point, and is shown (○).

In order to determine the timing and levels of *sigS* expression in *S. aureus* we created a *lacZ* reporter-fusion strain of SH1000. We cloned a 1405 bp fragment into the suicide vector pAZ106, which bears a promoterless *lacZ* cassette. This 1405 bp fragment runs from 945 basepairs upstream to 354 basepairs downstream of the *sigS* initiation codon. The possibility of additional promoter elements being present in this fragment was excluded by analysis of the *sigS* locus, revealing that SACOL1826 is located 199 bp from the *sigS* initiation codon, and is transcribed in a divergent orientation. This plasmid was first introduced into RN4220 before being transferred to SH1000. Analysis of this strain on TSA containing X-Gal revealed no blue coloration, even after incubation of up to 1 week. We then grew the SH1000 *sigS*-*lacZ* strain in liquid media for 3 days, removing aliquots at 1 hour intervals in order to assay for specific *sigS* expression. We found that even after 3 full days of growth, we could determine no expression of *lacZ* from the *sigS* reporter strain (Fig 5; maximum miller units were 19 at 52 h). The construct and mutant were independently regenerated 2 additional times to ensure that no unwanted genetic rearrangements had occurred with the plasmid, or plasmid bearing strains; yet in each case no *sigS* expression, as determined by β-Galactosidase activity, was detectable.

Studies of ECF-sigma factors in other organisms have demonstrated the induction of ECF-sigma factor expression in response to stress inducing compounds. Specifically, in one such study by Cao et al [59], an elegant disc-diffusion reporter-gene fusion method was employed to define conditions conducive to the expression of σ^W^ in *B. subtilis*. Thus we employed a similar technique using our *sigS*-*lacZ* fusion strain. TSA plates were overlayed with TSB top agar (0.7% w/v) seeded with exponentially growing SH1000 *sigS-lacZ* cells, and containing 40 µg ml^−1^ X-GAL. Sterile filter discs were overlayed onto these plates (3 per plate), before being inoculated with 10 µl of the following stress inducing chemicals: 30% H_2_0_2_, 80% cumene hydroperoxide, 500mM diamide, 2M methyl viologen, 1% menadione, 100mM plumbagin, 400 mg ml^−1^ pyrogallol, 100mM sodium nitroprusside, 10% SDS, 10% Triton X-100, 12M HCl, 6M NaOH, 95% ethanol, 2 mg ml^−1^ bacitracin, 2 mg ml^−1^ vancomycin, 5 mg ml^−1^ penicillin G and 20 mg ml^−1^ puromycin. Plates were incubated for 24 h at 37°C and screened for conditions conducive to σ^S^ expression as determined by a blue halo around the edge of the filter discs. Upon analysis we found that none of the chemicals tested resulted in the induction of σ^S^ expression, as determined by a lack of blue coloration on any of the test plates (data not shown).

### Investigating the effect of SACOL1828 on *sigS* expression

As referred to above, ECF-sigma factors are often encoded upstream of an ORF that specifies an anti-sigma factor. Whilst SACOL1828 would be an unusual anti-sigma factor, as it lacks any obviously membrane associated domains, we decided to assess its role on *sigS* expression. As σ^S^ seems to have a role in autoinducing its own transcription, it follows that if SACOL1828 were to inhibit the activity of the σ^S^ protein, then a SACOL1828 mutant would have higher *sigS* expression, as a result of an increase in free σ^S^ protein. Thus we generated a SACOL1828::*tet* mutant in SH1000, before transducing it with the *sigS*-*lacZ* reporter-gene fusion. The presence of both mutation and reporter-fusion were confirmed by PCR analysis, and the strain was assayed for β-Galactosidase activity. Much like that seen with the SH1000 *sigS*-*lacZ* fusion alone, we found that the inactivation of SACOL1828 had no effect on *sigS* expression. Indeed no β-Galactosidase activity was detectable in this strain even after 1 week of growth on TSA containing X-GAL. Because of the close proximity of the integration sites for the *sigS*-*lacZ* and SACOL1828 mutation we regenerated this strain via an alternative manner. Electrocompetent RN4220 SACOL1828::*tet* cells were prepared, and used as recipients for electroporation with the *sigS*-*lacZ* construct. Clones were analyzed for the presence of both the mutation and reporter-fusion by PCR analysis, before 2 representative clones were used to generate phage lysate using φ11. These lysates was then used to transduce SH1000, with transductants selected for on the basis of the resistances of either the mutation (tetracycline) or the reporter-fusion (erythromycin). Clones were screened by PCR to confirm the efficient constransduction of each marker. Again as with the *sigS*-*lacZ* reporter-fusion strain, the regeneration of this strain did not result in detectable β-Galactosidase activity.

### σ^S^ is required for the full virulence of *Staphylococcus aureus*

**Figure 6:**
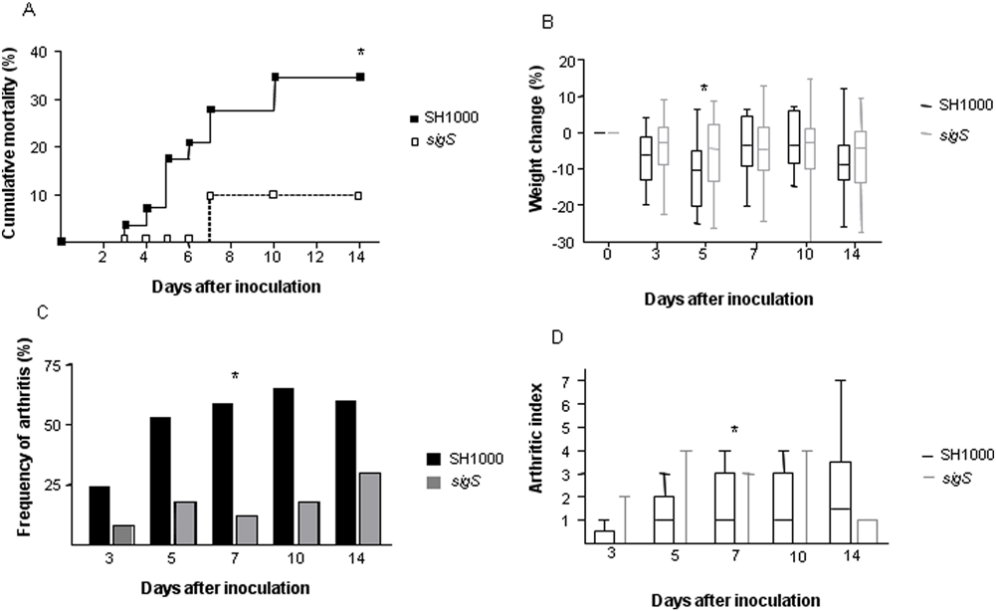
σ^S^ is required for the full virulence of *S. aureus* in a murine model of septic arthritis. (A), The cumulative mortality of mice (assessed by a log rank test, p<0.05). N = 30 per group. (B), Changes of body weight in the same mice as in A (*p<0.05 as compared using a Mann-Whitney U test.). (C), Frequency of clinical arthritis in mice inoculated with either wild-type *S. aureus* (SH1000) or its isogenic *sigS* mutant. The data from 2 separate experiments were pooled, n = 25 per group at day 3, n = 18 per group at days 5–10, and n = 10 per group at day 14. Statistical comparisons were performed using a chi-square test with Yates correction (*p<0.05). (D), Severity of clinical arthritis in the same mice as in C. Data is presented as medians (horizontal lines); inter-quartile ranges (bars) and ranges (error bars). An arthritic index was calculated by scoring all four limbs of each animal. Statistical comparisons were performed using a Mann-Whitney U test (*p<0.05).

As the number of ECF-sigma factors identified grows, attention is turning to their often considerable roles in bacterial virulence [46]. Therefore we studied the impact of σ^S^ on the virulence of *S. aureus* infection in a murine model of septic arthritis. Mice were intravenously inoculated with either the parental strain (SH1000) or its *sigS* mutant derivative. In initial experiments using higher doses of bacteria, ranging from 4.5×10^6^ to 8×10^6^ bacteria per mouse, infection with the *sigS* mutant gave rise to significantly less mortality when compared to animals infected with SH1000 (Fig. 6A). Data from 3 pooled experiments showed that only 3 out of 30 mice infected with the *sigS* mutant died during the 14 day experimental period, compared with 10 out of 30 mice infected with SH1000 (p<0.05). In addition, mice infected with the *sigS* mutant lost significantly less weight than mice infected with SH1000. At day 5 post-inoculation, mice infected with the *sigS* mutant had lost on average only 4.4% (−13.3% to +2.2%, IQR) of their body weight, whereas SH1000 infected mice had a median weight loss of 10.4% (−20.2% to −5%, IQR) (p<0.05, Fig. 6B). At later time points the weight changes in surviving animals were similar in the two groups, probably due to the markedly higher mortality of mice infected with SH1000. The development of clinical arthritis was significantly less frequent in mice infected with the *sigS* mutant, than in mice given the same dose of SH1000 (Fig. 6C). At 7 days post-inoculation with the *sigS* mutant only 2 out of 17 mice (12%) had clinically overt arthritis, as compared to 10 out of 17 mice (59%) infected with SH1000 (p<0.05). In addition, the severity of clinical arthritis at this time point was significantly reduced in the *sigS* mutant-infected mice, as compared to SH1000-infected mice (p<0.05, fig. 6D).

**Figure 7:**
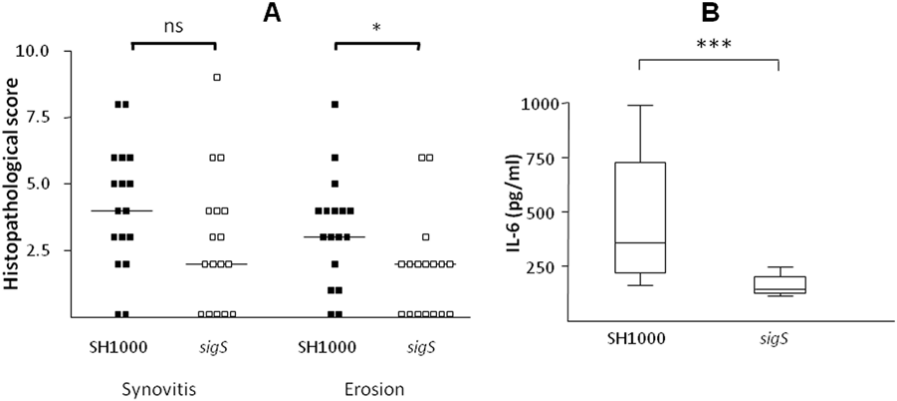
Analysis of the requirement for σ^S^ in *S. aureus* infection as measured via histopathological evaluation and mounted immune response analysis. (A), Histopathological evaluation of all limbs from mice 14 days post infection. The levels of synovitis and erosion (*p<0.05) were measured and mean scores are represented by vertical bars. (B), Serum IL-6 concentrations were determined for infected mice. All samples were run in triplicate. ***p<0.001.

Fourteen days after inoculation all limbs from the mice inoculated with 3×10^6^ to 4×10^6^ bacteria per mouse were subjected to histopathological evaluation. As shown in figure 7A, infection with the *sigS* mutant induced much less erosion of bone and cartilage as compared to infection with the parental strain (p<0.05). In addition, infection with the *sigS* mutant also induced somewhat milder joint inflammation than SH1000 (Fig 7A), although these results were not found to be statistically significant. The systemic immune responses of mice infected with the *sigS* mutant and SH1000 were also compared by analyzing the levels of the proinflammatory cytokine interleukin (IL)-6 in serum 14 days post-inoculation. Mice infected with 3×10^6^ bacteria of the *sigS* mutant had a median serum IL-6 concentration of 147 pg/ml (IQR 130–202 pg/ml; n = 10), which was markedly lower than the IL-6 concentration found in mice infected with SH1000, which had a median of 358 pg/ml (IQR 219–729 pg/ml; n = 10) (p<0.001, Fig 7B). Finally we investigated the ability of the strains to persist in host tissues, by determining the CFU/ml in kidney tissue homogenates. For this purpose, samples were taken from the kidneys 14 days after inoculation with 3×10^6^–4×10^6^ staphylococci per mouse. The *sigS* mutant clearly showed a reduced capacity to colonize host tissues, as it could not be detected in the kidneys of 6 out of 17 mice (35.3%). In contrast, growth of SH1000 was seen in the majority of infected animals, with only 2 out of 17 mice having negative kidney cultures (11.8%). The median number of staphylococci in the kidneys was 5×10^4^ (IQR 0–3.4×10^7^) bacteria after inoculation with the *sigS* mutant, as compared to 3.2×10^7^ (IQR 2.5×10^5^–1.3×10^8^) after inoculation with SH1000. Similar results were obtained after inoculation with higher doses of bacteria (data not shown).

## Discussion

*S. aureus* is a complex and versatile pathogen, which employs many different strategies in order to bring about its pathogenic response. It possess a diverse and wide-reaching network of regulatory elements that serve to fine-tune the coordinated expression of virulence determinants [13], [15], [20], [23], [24], so as to specifically bring about infection in a targeted manner. Additionally, there are a number of regulatory elements that contribute to the *S. aureus* virulence process, by controlling cellular physiology, and the adaptation to external conditions. The presumably facilitate both adaptation and proliferation in the harsh environment of the host [17], [18], [26], [31]. Such loci, whilst not always directly controlling virulence determinant production, are no less important to the virulence process, as they facilitate the rapid physiological switching that is a hallmark of *S. aureus*. This kind of responsiveness is commonly induced in other organisms by sigma factors, as they present a rapid and direct way of modulating stimulons in response to change. Rather unusually, *S. aureus* seemingly achieves its versatile and adaptive nature with only a limited selection of sigma factors. So far only three have been documented [27], [28], [29], and only one of these (σ^B^) has been shown to have a role in cellular adaptation and virulence [30], [31], [33]. The work presented in this current study demonstrates that an additional, and as yet uncharacterized, 4^th^ sigma factor (σ^S^) exists in *S. aureus*. σ^S^ appears to be a member of the ECF-family of sigma factors, and likely represents an important component of the stress and pathogenic responses of this organism.

Using biochemical approaches we have shown that σ^S^ is able to associates with core-RNAP, and initiate transcription from its own coding region. The autoregulation of ECF-sigma factor expression is a common hallmark of this family of regulators, and has been observed amongst a great many of their number [34]. Additionally, using a *sigS* mutant of *S. aureus*, we have shown that σ^S^ contributes to the protection against external stress, and plays a role in cellular fitness and survival. This is not unexpected, as the majority of ECF-sigma factors studied have been shown to function in the adaptation to stressful conditions [36]–[45]. In this study we present that σ^S^ is important for *S. aureus* cellular survival when faced with prolonged starvation, and extended exposure to elevated growth temperatures. Additionally a *sigS* mutant is seemingly less able to survive, at least initially, the attack on cell wall stability posed by Triton X-100. The observation of these phenotypes for σ^S^ is not out of keeping with other ECF-sigma factors, as a number are known to contribute to either heat shock responses and/or modulate cell wall stability [34].

On the other hand, using disc diffusion analysis, we were unable to find any increased sensitivity of the *sigS* mutant to a variety of chemical stresses, including those generating oxidative stress (H_2_0_2_, cumene hydroperoxide, diamide, methyl viologen, menadione, plumbagin, pyrogallol), nitric oxide stress (sodium nitroprusside), detergent stress (SDS, Triton X-100), acid and alkali stress (HCl, NaOH), alcohol stress (ethanol) and antibiotic stress (vancomycin, penicillin G, puromycin). Whilst this may appear unusual, given that a number of ECF-sigma factors in other organisms respond to these conditions, it is not entirely inexplicable. ECF-sigma factors are selectively induced in response to the specific stress that they are intended to combat. Thus it is likely the case that in *S. aureus*, σ^S^ is not the primary arbiter of adaptation to the stresses listed above. This is particularly pertinent to oxidative and antibiotic stress, as *S. aureus* has a variety of mechanisms by which to circumnavigate and survive these threats [60]–[69]. Therefore it is probably that the efforts exerted in the present study have yet to hit upon the specific condition to which σ^S^ is required to respond. Indeed it possible, given the data generated by our animal studies, that the specific stress(es) σ^S^ responds to are not ones that can be simulated *in vitro*, but are uniquely associated with the *in vivo* lifestyle of *S. aureus*.

With that said, it is apparent that *sigS* does present some benefit to the cell during *in vitro* growth. In our coculture studies, where the parent and mutant strain were grown together under a variety of conditions, it was clear that σ^S^ was a significant aid to the survival and fitness of *S. aureus*. When the SH1000 *sigS* mutant was forced to compete with its parental strain, it displayed significantly reduced abilities for growth and survival. This phenotype was only exacerbated during prolonged growth periods (7 days), or in the presence of external stressor compounds. This would tend to suggest that *sigS* presents a selective advantage to *S. aureus* cells both during standard growth conditions, as well as during times of starvation and/or stress. Therefore it would seem logical that σ^S^ is a valuable component for maintaining cellular harmony and stability, and as such likely represents an important mechanism by which *S. aureus* protects itself against the harsh environments encountered during growth.

Our transcription profiling studies of *sigS* turned up some interesting information regarding its expression. It appears that during growth under standard conditions, *S. aureus* SH1000 cells do not initiate expression from the *sigS* locus. Our studies, which were sampled every hour for 3 days, consistently revealed expression in the typically background range of 0–1 Miller units. Only in 2 instances during growth did we detect anything higher than these values (32–36 h, and 48–52 h), and even then maximal expression was only 19 Miller units. We have generated a number of *lacZ* reporter-fusion strains in a variety of *S. aureus* backgrounds [30], [70]–[72] (unpublished data), and have never seen a strain that displays such limited expression under specific analysis. Even upon the analysis of apparently very lowly expressed genes (e.g. SH1000 *ssp*-*lacZ* fusion), which display little to no blueness on TSA X-gal plates, we routinely observe expression units in the hundreds [30]. With this in mind, and given the length of our transcription experiment, it is likely that even these 2 windows of minor expression may be the result of something other than actual induction of the *sigS* operon (e.g. cellular lysis). Therefore, as asserted above, this would tend to suggest that *sigS* is not expressed in SH1000 during growth under standard conditions.

This is certainly an unusual observation, but as ECF-sigma factors are commonly inducibley expressed in response to stress conditions, it perhaps not surprising. Indeed, analysis of the ECF-sigma factors of *B. subtilis* provides similar examples of transcriptional regulation. For example it has been reported that transcription of the ECF-sigma factor σ^Z^ from *B. subtilis* is undetectable during growth in rich and minimal media [73]. Further, specific analysis of *B. subtilis* ECF-sigma factor expression, conducted by Asai et al [74], revealed that the expression of σ^V^, σ^Y^ and σ^YlaC^, in addition to σ^Z^, were all equally low, and barely detectable during growth under standard conditions. In a study aimed at defining conditions conducive to σ^W^ expression in *B. subtilis*, by Cao et al [59], an elegant disc-diffusion reporter-gene fusion method was employed. Cells bearing a *sigW*-*lacZ* fusion were grown on LB agar containing X-GAL, and overlayed with filter discs containing a variety of antibiotics. Using this approach, chemicals conducive to σ^W^ expression yielded a halo of blue around the edge of the filter disc. We employed just such an approach with our SH1000 *sigS*-*lacZ* fusion, using the chemicals previously tested in sensitivity assays with the SH1000 *sigS* mutant. Perhaps unsurprisingly, we found none of the chemicals tested resulted in an increase in σ^S^ expression. This would tend to add further weight to our assertion that in the present study have yet to hit upon the specific condition to which σ^S^ is induced in *S. aureus*.

Further transcriptional analysis focused on the role of SACOL1828 on σ^S^ expression. As referred to above, ECF-sigma factors are often encoded upstream of an ORF that specifies an anti-sigma factor. As σ^S^ seems to have a role in autoinducing its own transcription, it follows that if SACOL1828 were to inhibit the activity of the σ^S^ protein, then a mutation in SACOL1828 would have higher *sigS* expression as a result of more free and active σ^S^ protein. Indeed similar approaches have been used to analyze the putative anti-sigma factors of *B. subtilis* ECF-sigma factors, including σ^YlaC^ and σ^X^ [75], [76]. Our analysis found that inactivating SACOL1828 did not result in an increase in *sigS* expression, as would have been predicted if SACOL1828 were to function as an anti-sigma factor. We suggest, however that this observation may be explained by the apparent lack of *sigS* expression in SH1000. If, as we find, there is little to no *sigS* expression in SH1000 during growth under standard conditions, then it follows that there is little to no σ^S^ protein present in the cell. Therefore the inactivation of a σ^S^ anti-sigma factor would not bring about the predicted snowballing of *sigS* expression, resulting from free σ^S^ protein being able to auto-stimulate its own transcription. Thus it appears that further investigation is required before we can specifically determine whether SACOL1828 plays any role in the regulation of σ^S^ activity.

The most striking, and indeed important, role we have defined for σ^S^ is its role in the virulence of *S. aureus*. Using our murine model of septic arthritis infection we have demonstrated that in each of the tests applied, to determine the extent and severity of disease, *S. aureus* cells lacking a functional *sigS* gene were significantly impaired in their ability to establish and maintain infection. Mice infected with *S. aureus* in this model lose weight, undergo extreme destruction of joints, bone and cartilage, and ultimately die. However those mice infected with the *sigS* mutant lost significantly less weight, developed septic arthritis at considerably lower levels, and most tellingly, had considerably increased survival rates. In addition, our studies of mounted immune responses by infected mice reveal that those animals infected with the *sigS* mutant had significantly lower levels of IL-6, indicating only a very weak immune response to the invading pathogens. Finally, a major hallmark of septic arthritis is systemic dissemination, moving from the site of infection into the kidneys. Our analysis reveals that mice infected with the parental strain possessed large numbers of *S. aureus* cells in the kidneys of infected mice. However when the same analysis was conducted with the *sigS* mutant it was apparent that strains of *S. aureus* lacking a functional *sigS* gene were far less able to undergo systemic dissemination. Collectively, the virulence data that we present speaks very strongly to the importance of σ^S^ in the ability of *S. aureus* to cause disease, a fundamental cornerstone of its innate behavior.

From our investigations presented here we have demonstrated that σ^S^ is important for the *S. aureus* stress response, aiding in the protection against unfavorable conditions. In addition we have shown that it is vital for the infectious nature of *S. aureus*, as a *sigS* mutant is attenuated in virulence in a murine model of septic arthritis infection. However the specific and mechanistic role of σ^S^ in *S. aureus* biology remains unknown. It is unlikely; thought not impossible, that σ^S^ wields its role via direct regulation of virulence determinant expression. A more probable scenario is that σ^S^, as with other ECF-sigma factors, is responsible for sensing and responding to discrete external cue(s); and changing *S. aureus* gene expression profiles so as to protect the cell. It is the current and future purpose of our laboratory to explore and develop an understanding of the role of σ^S^, which will doubtlessly further our knowledge of this important human pathogen and its disease causing abilities.

## Materials and Methods

### Bacterial strains, plasmids and growth conditions

The *S. aureus* and *E. coli* strains, along with the plasmids used in this study are listed in Table 2. *E. coli* was grown in Luria-Bertani (LB) medium at 37°C. *S. aureus* was grown in 100 ml TSB (1∶2.5 flask/volume ratio) at 37°C with shaking at 250 rpm, unless otherwise indicated. For growth analysis experiments, overnight cultures were inoculated into fresh media to an OD_600_ of 1.0 and allowed to grow for 3 hours. These cultures were then in turn used to inoculate fresh TSB to an OD_600_ of 0.01, and these were used as test cultures. CFU/ml counts were determined by the serial dilution of test-cultures onto TSA, followed by enumeration after overnight growth. All CFU/ml values represent the mean from three independent experiments. When required antibiotics were added at the following concentrations: ampicillin 100 µg ml^−1^ and tetracycline 12.5 µg ml^−1^ (*E. coli*); tetracycline 5 µg ml^−1^, erythromycin 5 µg ml^−1^and lincomycin 25 µg ml^−1^ (*S. aureus*). Where appropriate, X-GAL was added to media at a concentration of 40 µg ml^−1^.

**Table 2:** Strains, plasmids and primers used in this study. Where applicable restriction sites are underlined.

Strain, Plasmid or Primer	Genotype or Description	Reference/Source
***E. coli***		
DH5α	φ80 Δ( (*lacZ*)*M15* Δ( (*argF-lac*)*U169 endA1 recA1 hsdR*17 (r_K_^−^m_K_^+^) *deoR thi*-1 *supE*44 *gyrA*96 *relA*1	78
Tuner	F^−^ *ompT hsdS*_B_ (r_B_^−^ m_B_^−^) *gal dcm lacY1*(DE3) pLysS (Cam^R^)	Novagen
***S. aureus***		
RN4220	Restriction deficient transformation recipient	Lab Stocks
SH1000	Functional *rsbU* derivative of 8325-4 *rsbU* ^+^	30
LES55	SH1000 *sigS*::*tet sigS* ^−^	This Study
LES56	SH1000 SACOL1828::*tet* SACOL1828^−^	This Study
LES57	SH1000 pAZ106::*sigS*-*lacZ sigS* ^+^	This Study
LES58	RN4220 SACOL1828::*tet* SACOL1828^−^	This Study
LES59	RN4220 SACOL1828::*tet* pAZ106::*sigS*-*lacZ sigS* ^+^ SACOL1828^−^	This Study
**Plasmids**		
pAZ106	Promoterless *lacZ erm* insertion vector	77
pET24d	6His-tag overexpression vector	Novagen
pLES200	pET24d containing a 470bp *sigS* fragment	This Study
pLES201	pAZ106 containing a 2.3kb *sigS* fragment	This Study
pLES202	pAZ106 containing a 2.2kb SACOL1828 fragment	This Study
pLES203	pLES201 containing a tetracycline cassette within *sigS*	This Study
pLES204	pLES202 containing a tetracycline cassette within SACOL1828	This Study
pLES205	pAZ106 containing a 1.4kb *sigS* fragment	This Study
**Primers**		
OL-281	ACTGGATCCCAGTTGCAGATGCATCTCTCC	
OL-282	AGCTAGGCATGCCAAGTCTATCTGGCGTAC	
OL-285	ACTGGATCCGACCATCACGATACATCA	
OL-286	CTTCACTGACAACTATGCCG	
OL-287	GCGATTACATTCTAGAAGTTCC	
OL-288	GGAACTTCTAGAATGTAATCGC	
OL-293	ATGGAATTCGTTTGAGCCATAGTCTTTCTC	
OL-297	ATGGAATTCCTAATTAAAATTATGTTGGCATTTA	
OL-387	GATGAGTATTATCAACTACTCTTG	
OL-389	ATGACCATGGTGAAATTTAATGACGTATAC	
OL-390	ATGACTCGAGATTAAAATTATGTTGGATTTTACGC	
OL-429	TATCAACTACTCTAGATAAAAATGTGGC	
OL-430	GCCACATTTTTATCTAGAGTAGTTGATA	
OL-522	ATGTCTAGAGAGTAATGCTAACATAGC	
OL-523	ATGTCTAGACCCAAAGTTGATCCCTTAACG	

### Overexpression and Purification of σ^S^

The 470bp *sigS* coding region was PCR generated using primer pair OL-389/OL-390 and cloned into the *E. coli* overexpression vector pET24d (Novagen) to create pLES200. The plasmid was subjected to DNA sequence analysis (UGA core facility) to ensure that the coding region was generated without mistake. This plasmid was purified from *E. coli* DH5α and transferred to the *E. coli* expression host Tuner (Novagen). Cells were grown at 37°C (in LB supplemented with 34 mg/l chloramphenicol and 30 mg/l kanamycin) before the induction of protein expression with 100 mM IPTG at an OD_600_ of 0.5. The culture temperature was then reduced to 30°C and growth was permitted for a further 4–5 h with vigorous agitation. Cells were harvested by centrifugation (10 min, 4,500 g), resuspended (Buffer A: 50 mM Tris-HCl pH 8.0, 100 mM NaCl, 50 mM imidazole) and disrupted by sonication. Soluble protein fractions, collected by centrifugation (30 min, 14,000g, 4°C), were applied to a Chelating Sepharose (Amersham) Ni^2+^ affinity column (1.5cm×1.6cm). To ensure saturated binding of the recombinant σ^S^ to the matrix, samples were circulated through the column for 2.5 h using the Akta Explorer system (Amersham), and then washed extensively with Buffer A until the OD_280_ of the eluate dropped to a baseline reading. Recombinant σ^S^ was eluted from the column in a stepwise manner with buffer A containing imidazole at 140, 320 and 500 mM concentrations. Fractions eluted at 320 mM were pooled and lyophilized in order to concentrate the purified recombinant protein. This was then resuspended in water, desalted (HiTrap desalting column, Amersham) by buffer exchange (20 mM Tris-HCl pH 8.0, 20 mM NaCl) and re-lyophilized. Protein purity was assayed by SDS-PAGE, yielding a single band with a molecular mass of 19 kDa. The presence of the 6His-Tag in recombinant σ^S^ was confirmed by Western Blot with anti-HisTag antibodies (Roche).

### σ^S^-Core RNAP Association Experiments

200 µl of anti-His-tag antibodies conjugated to agarose beads (Sigma) were washed thoroughly (20 mM Tris-HCl pH 8.0, 10 mM NaCl) and incubated with 250 µl of 0.2 mg/ml recombinant σ^S^ at room temperature for 3 h. The resin was washed thoroughly with 20 mM Tris-HCl pH 8.0, 10 mM NaCl and TBS-Tween, before adding 20 µl of core-RNAP at 1U µl^−1^ (Epicentre). Samples were then incubated for 2(h at room temperature followed by extensive washing. After adding SDS-PAGE sample buffer, samples were boiled and centrifuged (10(min, 16 000(g), and the supernatant subjected to SDS-PAGE.

### Transcription Run-Off Experiments

0.25 µg of core-RNAP (Epicentre) was preincubated with 1 µg of σ^S^ in transcription buffer [30mM Tris-HCl (pH 8.0), 10mM MgCl2, 100mM KCl, 1mM DTT], at 4°C for 15mins. After this time, 1 µg of a 1168 bp DNA-fragment (PCR generated using primer OL-281/OL-297), containing the *sigS* coding region and 945 bp of upstream sequence, was added to the σ^S^-core-RNAP complex, and further incubated at 37°C for 15mins. Transcription was initiated by the addition of 2.5 µM rNTPs, and transcription was allowed to proceed for 30 mins at 37°C. After this time the mixture was cleaned via 2 acid-phenolchloroform extractions (to remove DNA contamination), followed by isopropanol precipitation. The purified mRNA transcript was then subjected to a 1-step RT-PCR reaction using primer pair OL-387/OL-293 (104 bp from the initiation codon to 137bp from the termination codon, with a target fragment size of 274bp) and the Superscript III enzyme (Invitrogen). This experiment was repeated, omitting either purified σ^S^ or core-RNAP as controls. RT-PCR reactions were resolved on a 2% agarose gel and visualized using a BioDocIt Device (UVP).

### Construction of the *sigS* and SACOL1828 mutant strains

A plasmid for the mutagenesis of *sigS* was constructed by PCR amplification. Two approximately 1kb fragments were PCR generated surrounding the *sigS* coding region (1 located upstream, primer pairs OL-281/OL-430; and 1 located downstream, primer pairs OL-282/OL-429). Primers OL-429 and OL-430 are identical, but divergent to each other, and each contain base pair mismatching, converting the wild type sequence of TCAAGC (∼100bp from the *sigS* Met) to TCTAGA, an XbaI restriction recognition site. These fragments were purified and used together as the template for a further round of PCR with primer pair OL-281/OL-282. The resultant 2.3 kb DNA fragment was digested with BamHI and SphI, and cloned into the suicide vector pAZ106 [77] to generate pLES201, using standard cloning techniques [78].

A plasmid for the mutagenesis of SACOL1828 was constructed in a similar manner with the following exceptions. The two approximately 1kb fragments were generated using primer pairs OL-285/OL-288, and OL-286/OL-287. Primers OL-287 and OL-288 are identical, but divergent to each other, and contain mismatching that converts the wild type sequence of TCTTAA (∼100bp from the SACOL1828 Met) to a TCTAGA XbaI site. These fragments were used as the template for further PCR using primer pair OL-285/OL-286. This 2.2 kb DNA fragment was digested with BamHI and SphI and cloned into pAZ106 to generate pLES202 .

The novel XbaI sites in pLES201 and pLES202 were then used as a target sites for the insertion of a tetracycline resistance cassette, generated from pDG1515 [79] using primer pair OL-522/OL-523. The XbaI digested cassette was cloned into pLES201 and pLES202, yielding pLES203 (*sigS*) and pLES204 (SACOL1828). These were then used to transform electrocompetent *S. aureus* RN4220, according to the method of Schenk and Ladagga [80], with clones selected for on the basis of tetracycline and erythromycin resistance. Integrants were confirmed by PCR analysis (data not shown) and used as donors for the transduction of *S. aureus* strain SH1000 using phage φ11. Transductants were selected for their resistance to tetracycline (indicating the presence of the cassette) and sensitivity to erythromycin (indicating loss of the plasmid and associated functional copy of *sigS* or SACOL1828), before being confirmed by PCR analysis. This created strains LES55 (*sigS*) and LES56 (SACOL1828).

### Construction of a *sigS-lacZ* reporter-fusion strain

The putative promoter region of *sigS* was amplified as a 1.4 kb PCR fragment using primer pair OL-281/OL-293 (Table 2). The purified DNA fragment was digested with BamH1 and EcoRI and cloned into similarly digested pAZ106. *S. aureus* RN4220 was transformed with the resulting plasmid, pLES205, and integrants were confirmed by PCR analysis. A representative clone was then used to transduce *S. aureus* SH1000 using φ11, with clones again confirmed by PCR analysis. This created strain LES57 (*sigS*-*lacZ*).

### β-Galactosidase assays

Levels of β-Galactosidase activity were measured as described previously [71]. Fluorescence was measured using a Bio-Tek Synergy II plate reader, with a 0.1 sec count time, and calibrated with standard concentrations of MU (4-methyl umbelliferone). One unit of β-Galactosidase activity was defined as the amount of enzyme that catalyzed the production of 1 pmol MU min^−1^ OD_600_ unit^−1^. Assays were performed on duplicate samples and the values averaged. The results presented here were representative of three independent experiments that showed less than 10% variability.

### Disc-Diffusion Assays

Disk diffusion sensitivity assays were performed as follows: 5 ml of TSB top agar (0.7%, wt/vol) was seeded with 5 µl of exponentially growing strains of *S. aureus*, and used to overlay TSA plates. Sterile filter disks were placed in the centre of the overlayed plates, and 10 µl of the test chemicals was applied at the following concentrations: 30% H_2_0_2_, 80% cumene hydroperoxide, 500mM diamide, 2M methyl viologen, 1% menadione, 100mM plumbagin, 400 mg ml^−1^ pyrogallol, 100mM sodium nitroprusside, 10% SDS, 10% Triton X-100, 12M HCl, 6M NaOH, 95% ethanol, 2 mg ml^−1^ bacitracin, 2 mg ml^−1^ vancomycin, 5 mg ml^−1^ penicillin G and 20 mg ml^−1^ puromycin. This technique was also adapted for transcription profiling using the SH1000 *sigS*-*lacZ* strain. In this situation 5 µl of exponentially growing *sigS*-*lacZ* cells was seeded into 5 ml of TSB top agar (0.7%, wt/vol) containing X-GAL (40 µg ml^−1^). This was then used to overlay TSA plates before sterile filter discs (3 per plate) were placed on top of the agar overlay. Filter discs were then seeded with 10 µl of the same stress inducing chemicals listed above.

### Cell Wall Lysis Experiments

Lysis kinetics using lysostaphin and Triton X-100 were performed as described previously [81]. Penicillin G lysis was performed as described by Fujimoto & Bayles [82].

### Coculture experiments

SH1000 and the SH1000 *sigS* mutant were grown in competitive culture experiments as described previously by Doherty et al [83]. Briefly, both strains were grown separately for 18h in TSB under standard conditions. Cells were harvested by centrifugation, washed with PBS and used to inoculate fresh TSB with an inoculation ratio of 1∶1. These ratios were confirmed by retrospective viable counts of the starting inoculum in triplicate. Cultures were incubated at 37°C for the times specified and viable counts were again determined in triplicate. These experiments are facilitated by the tetracycline resistance cassette used to mark the *sigS* mutant. Therefore plating dilutions of the coculture on both TSA, and TSA containing tetracycline, allows derivation of exact colony counts for each strain, and thus calculation of the competitive index (CI).

### Experimental models of *S. aureus* sepsis and arthritis

Female NRMI mice, 6 to 8 weeks old, were purchased from B & K Universal AB (Sollentuna, Sweden) and kept in the animal facility of the Department of Rheumatology and Inflammation Research, Göteborg University. *S. aureus* strain SH1000 and its isogenic *sigS* mutant were cultured on horse-blood agar plates at 37°C for 24 hours, harvested, washed in PBS and resuspended in PBS supplemented with 10% dimethyl sulfoxide and 5% bovine serum albumin. Aliquots of bacterial suspensions with a known CFU/ml, as determined by viable counts, were stored at −20°C. Before inoculation, bacterial cultures were thawed, washed once with PBS and diluted in PBS to the desired concentration. In five independent experiments mice were inoculated intravenously with 200 µl of bacterial suspension in declining bacterial doses (8×10^6^, 6×10^6^, 4.5×10^6^, 4×10^6^, and 3×10^6^ CFU/mouse). Viable counts of the inoculum were performed in each experiment to confirm the accuracy of each dose. Mice were individually monitored for up to 14 days by an observer (CL) blinded to the identity of the experimental groups for general appearance, weight change, mortality, and the development of arthritis, before being sacrificed. Clinical arthritis, defined by visible erythema and/or swelling of at least one joint, was scored from 0 to 3 for each limb (1, mild swelling and/or erythema; 2, moderate swelling and erythema; 3, marked swelling and erythema). An arthritic index was generated by adding the scores for each limb of a given animal. Histopathological evaluations of the limbs were performed after routine paraformaldehyde fixation, decalcification, paraffin embedding, and hematoxylin and eosin staining. Tissue sections were evaluated for synovitis and joint destruction by an observer (CL) blinded to the identity of the groups. Synovitis and cartilage/bone destruction were scored separately as 0, none; 1, mild; 2, moderate; and 3, for severe synovial hypertrophy and joint damage. The sum of all of the limbs was used to calculate a histopathology score.

Bacterial persistence in host tissues was evaluated by aseptically removing the kidneys, homogenizing them and performing viable counts after serial dilution in PBS. The CFU/ml were determined after 24 hours of cultivation on horse blood agar plates. Serum IL-6 concentrations were determined as previously described, using a bioassay in which the murine hybridoma cell line B9 is dependent on IL-6 for growth, [84]. All samples were run in triplicate, and the statistical evaluations of weight change and severity of clinical and histopathological arthritis between groups was performed using a Mann-Whitney U test. A chi-square test was used for comparison of frequency of clinical arthritis between groups, whilst the comparison of mortality was done by a log rank test. A p-value <0.05 (after Bonferroni correction for multiple comparisons) was deemed to indicate statistically significant differences.
